# Intersections between COVID-19 and socio-economic mental health stressors in the lives of South African adolescent girls and young women

**DOI:** 10.1186/s13034-022-00457-y

**Published:** 2022-03-26

**Authors:** Zoe Duby, Brittany Bunce, Chantal Fowler, Kate Bergh, Kim Jonas, Janan Janine Dietrich, Darshini Govindasamy, Caroline Kuo, Catherine Mathews

**Affiliations:** 1 Health Systems Research Unit, South African Medical Research Council, Francie van Zijl Drive, Parow Valley, Tygerberg, Cape Town, South Africa; 2 Division of Social and Behavioural Sciences in the School of Public Health and Family Medicine, University of Cape Town, Cape Town, South Africa; 3 Institute for Global Sustainable Development, University of Sheffield, Sheffield, UK; 4 Perinatal HIV Research Unit (PHRU) and African Social Sciences Unit of Research and Evaluation (ASSURE), School of Clinical Medicine, Faculty of Health Sciences, University of the Witwatersrand, Johannesburg, South Africa; 5 Department of Behavioral and Social Sciences, Brown University School of Public Health, Providence, RI USA

**Keywords:** COVID-19, Lockdown, Adolescent girls and young women, Mental health, South Africa

## Abstract

**Background:**

In contexts where poverty and mental health stressors already interact to negatively impact the most vulnerable populations, COVID-19 is likely to have worsened these impacts. Before the COVID-19 pandemic, adolescent girls and young women (AGYW) in South Africa already faced intersecting mental health stressors and vulnerabilities. It is critical to understand how additional challenges brought on by COVID-19 have intersected with existing vulnerabilities and mental health risks AGYW faced, particularly given the intersections between psychological distress and increased risk behaviours that impact sexual and reproductive health. We aimed to examine socio-economic and mental health impacts of COVID-19 on South African AGYW in order to understand how additional challenges brought on by COVID-19 have intersected with existing challenges, compounding AGYW vulnerabilities.

**Methods:**

Using qualitative and quantitative methods, framed by the syndemic theory, we examined the intersections between mental health and the COVID-19 epidemic amongst AGYW in six districts of South Africa characterised by high rates of HIV, teenage pregnancy and socio-economic hardship. Between November 2020 and March 2021 we conducted a cross-sectional telephone survey with 515 AGYW, and in-depth interviews with 50 AGYW, aged 15 to 24 years.

**Results:**

Our findings reveal how COVID-19 restrictions led to increased experiences of stress and anxiety. Poor mental health was compounded by strained family relationships, increased fear of domestic violence, household unemployment, economic stress and food insecurity. Respondents described feelings of boredom, frustration, isolation, loneliness, fear and hopelessness. However, despite the multitude of challenges, some AGYW articulated emotional resilience, describing ways in which they coped and retained hope.

**Conclusion:**

Various psycho-social risk factors already disproportionally affect the mental health of AGYW in these communities; the COVID-19 pandemic intersects with these pre-existing social and environmental factors. Understanding strategies AGYW have used to positively cope with the uncertainty of COVID-19 amongst an array of pre-existing mental health stressors, is key in informing efforts to respond to their needs. Multisectoral interventions are needed to address the drivers of poor mental health among AGYW, and bolster healthy coping mechanisms; interventions seeking to mitigate the mental health impacts on this vulnerable population need to be responsive to the unpredictable pandemic environment.

## Background

In public health literature, the syndemic theory provides a framework for understanding how overlapping and co-occurring risk factors situated in a specific social context, cluster to create intersecting epidemics which combine to enhance vulnerability [1]. In contexts where poverty and mental health problems already interact to negatively impact mental health of the most marginalised and vulnerable populations, COVID-19 is likely to have heightened and worsened these impacts. This is particularly the case in a country like South Africa, where there is considerable psychiatric morbidity, limited mental healthcare infrastructure, and high rates of poverty and unemployment [2, 3]. Poor mental health, including depressive disorders and stress, contributes significantly to the burden of disease in South Africa, and other parts of sub-Saharan Africa [4, 5]. Of all African countries, South Africa has had the highest number of COVID-19 cases (34% of all cases on the continent), and accounts for 43% of all reported COVID-19-related deaths on the continent (South Africa’s COVID-19 related deaths are estimated to be around 300,000^1^) [6]. The country’s under-resourced public health system, already overburdened by infectious and chronic diseases, was characterised by constrained access to mental health care services even prior to the pandemic [7, 8].

Given the impact of previous large-scale ‘disasters’ on mental health stressors resulting from drastic social control measures, it is expected that the COVID-19 global pandemic has similarly negatively impacted on mental health [9]. COVID-19 related mental health stressors come against a backdrop of a pre-existing public health crisis in South Africa due to a gap in mental health service provision and constrained access to mental health care services, with COVID-19 related restrictions adding to the burden of mental health in the country [8, 10]. Little is known about the mental health impacts of the COVID-19 pandemic and lockdown regulations in South Africa and globally, but growing evidence suggests worsening mental health and increased levels of distress, emotional isolation, depression and anxiety amongst adolescents and young people, against a backdrop of exacerbated psycho-social risk factors such as bereavements, disruptions in education and life routines, and worry about the future [11, 11, 12]. Emerging evidence from COVID-19 Mental Disorders Collaborators^2^ suggests that the prevalence of major depressive disorder and anxiety disorders and during the COVID-19 pandemic have drastically increased, with South Africa ranking amongst the countries globally with the most marked increases.

Socio-economic factors are key drivers of stress, depression and anxiety, as well as other structural determinants of mental health including housing and food security [14, 15]. Poverty, low household income and food insecurity (defined as uncertainty about and ability to obtain food, and being forced to compromise on quality/quantity of food consumed due to lack of money or resources) are associated with heightened vulnerability to experiencing poor mental health, including depression and anxiety disorders [9, 15, 16]. In South Africa, the majority of the population are already economically vulnerable, and disproportionately affected by unemployment and poverty. The COVID-19 crisis has worsened unemployment, caused disruptions in domestic food supply chains, and created widespread food insecurity [14].

Since adolescence is the period of an individual’s life in which mental health difficulties are most likely to develop, and particularly for adolescent girls and young women (AGYW) in South Africa, who are more susceptible to depressive symptoms than their male counterparts, understanding the effects of COVID-19 on the mental health of South African AGYW is imperative [1, 12]. In addition to neurological, hormonal, and physical changes associated with puberty, changes in adolescents’ social environments play a key role in the onset of mental health challenges during this time [17]. The drastic changes in life routine and social environments, including social distancing, worry for families, unexpected bereavements, disruptions in education, confinement restrictions, and concern about the future brought about as a result of the pandemic, are likely to have disproportionately affected adolescents, given how crucial this period is in the development of social connections [12, 18]. Adolescents growing up in the context of socio-economically adverse communities are faced with a range of additional psychosocial and health risks that may evoke stress and negatively affect their mental health; these risks include exposure to HIV and other stressors [12].

The COVID-19 pandemic’s interactions with pre-existing mental health stressors offers an important opportunity to understand coping mechanisms. Coping strategies are an individual’s efforts to manage their distress, although they are not always effective at doing so. Particular positive coping strategies (e.g., acceptance, seeking support) are associated with greater psychological health than other, more maladaptive strategies [19]. Understanding what strategies young people have been using to cope with the uncertainty of COVID-19 is key to inform efforts to respond to their needs. Psychological resilience is one important aspect of how individuals cope with adversity; resilience refers to an individual’s ability to withstand set-backs and adapt positively in the face of adversity, combined with perceived social support can be protective against negative mental health outcomes [25]. An individual’s ability to tolerate uncertainty will likely influence the way they respond and cope with a situation [19].

Negative mental health impacts are expected to be particularly felt amongst already vulnerable and marginalised populations in under-resourced settings [18, 20]. People whose human rights are least protected, such as AGYW in the poorest communities, are likely to be disproportionately affected by the devastating economic and social consequences of the COVID-19 pandemic, further restricting their access to health services, social protection, and other support services [21, 22]. Given the fast changing context of the COVID-19 epidemic in South Africa, government-mandated restrictions, and adaptations to social, educational and health policies, understanding the psychological and social impacts of the COVID-19 pandemic and associated lockdown restrictions on adolescents and young people in South Africa is critical to inform the rapid development of policies and age and developmentally-appropriate interventions to mitigate mental health problems and provide tailored support during and after the pandemic [23, 24]. This is particularly crucial for those that already experience vulnerability, in order to inform policy and intervention design and to best mitigate further negative impacts and offer vulnerable populations the necessary support [25].

Before the COVID-19 pandemic, AGYW in South Africa already faced intersecting mental health stressors and vulnerabilities including insufficient social support, economic insecurity, and gendered and age-related vulnerabilities [1]. As yet, little is understood about the impacts that the COVID-19 pandemic and the lockdown implemented by the South African government have had on South African AGYW’s mental health, but in light of emerging evidence, researchers have called for the scientific community and policymakers to consider the mental health impacts of the COVID-19 pandemic [11, 13]. There is a need to understand the differential impacts of the pandemic on women and girls, to ensure that gender dimensions are not overlooked in on-going interventions [21]. We used quantitative and qualitative methods to examine socio-economic and mental health impacts of COVID-19 on South African AGYW in order to understand how the additional challenges brought on by COVID-19 have added to and intersected with existing challenges, compounding AGYW vulnerabilities and risks.

### Situating this study

Due to the drastic and rapidly changing environment globally and in South Africa in response to the evolving COVID-19 situation, viral outbreaks, and waves of infection, it is critical to situate the research activities temporally. In response to the emerging pandemic and to curb the spread of the virus, in March 2020 the South African government introduced an initial “lockdown”, comprising country-wide restrictions on freedom of movement. In May 2020, there was an initial easing of restrictions, followed by further easing of restrictions in June [26]. In July 2020, amidst rising infections, tighter restrictions were again imposed. In September 2020 restrictions eased again, after which followed a relative period of calm and a sense of life returning to normalcy. Following another resurgence of infections, the Minister of Health announced that the country had entered a second wave, and tighter restrictions were imposed during December 2020. Subsequent lockdowns of varying severity were imposed in response to waves of COVID-19 infection in the country. Interacting with pre-existing mental health stressors, particularly in socio-economically and resource constrained settings, such as informal settlements, social distancing measures and business closures, introduced to flatten the infection curve, significantly impacted most people’s usual activities, routines and livelihoods [10]. While such restrictions have an epidemiological rationale based on slowing down the spread of the virus, it is critical to understand how they affected the daily lived reality of South Africans [27, 28]. Data collection for the present study was conducted between November 2020 and March 2021.

## Methods

In this study we examined the intersections between mental health and the COVID-19 epidemic amongst AGYW in South Africa. We conducted a survey and qualitative interviews, nested within a study evaluating an intervention for AGYW in South Africa.^3^ Data collection took place between November 2020 and March 2021, in six South African districts, spanning six provinces of South Africa: Klipfontein, City of Cape Town (Western Cape), King Cetshwayo (KwaZulu Natal), Ehlanzeni (Mpumalanga), Bojanala (North West), Nelson Mandela Bay (Eastern Cape), and Dihlabeng, Thabo Mofutsanyana (Free State). The districts and sub-districts from which the samples were drawn are diverse, some urban, some peri-urban, and some rural. A commonality across all six study communities is that they are situated in areas characterised by high HIV prevalence and high rates of teenage pregnancy (Table 1). In South Africa, health and socio-economic indicators vary hugely by province, but also within provinces, between districts and sub-districts. For example, Klipfontein is located in the City of Cape Town in the Western Cape, which is a relatively wealthy and well-resourced province. However, Klipfontein sub-district is a poorly resourced and deprived community within a wealthy city, and has an antenatal HIV prevalence of 23.4%, compared to 10.3% in the neighbouring Southern sub-district [29]. Some of the other study sites are rural districts within relatively poor provinces, for example Thabo Mofutsanyana in the Free State.

**Table 1:** HIV prevalence among women attending antenatal (ANC) clinics in HERStory2 study sites

Study site district/sub-district	Province	ANC HIV prevalence (%)
Klipfontein sub-district, City of Cape Town district	Western Cape	23.4^*^
King Cetshwayo district	KwaZulu Natal	35.0
Ehlanzeni district	Mpumalanga	36.8
Bojanala district	North West	31.5
Nelson Mandela Bay district	Eastern Cape	31.4
Dihlabeng sub-district, Thabo Mofutsanyana district	Free State	32.1

Source: The 2019 National Antenatal Sentinel HIV Survey, South Africa, National Department of Health; * Source: Essel, 2014

### Sampling

Samples for both quantitative and qualitative components were drawn using a de-identified version of the intervention’s monitoring database, which included a comprehensive anonymised list of the 127,951 AGYW beneficiaries. In the sampling frame, beneficiaries were stratified by district and age group, and for the younger age group, by whether they were in school. In the target sample, we doubled the number of AGYW in the younger age group (compared with the older age group), as we expected that only approximately 50% of them would have ever had sex (most of the key outcomes in the evaluation in which this study was nested, were only relevant to those who had ever had sex).

The sample size allowed for a 50% non-response or refusal rate. However, the implementers were unable to contact many of the sampled beneficiaries; reported reasons for this included that phone calls were unanswered, phone numbers were not valid, or there was no phone number in the records. The proportion of the sampled beneficiaries who were uncontactable varied by district from 32.7% to 74.6%. Phone numbers of sampled AGYW were obtained, with their knowledge and consent. When the implementers successfully contacted the sampled beneficiary, most beneficiaries agreed to be called by a study team member. Between 0 and 10% of sampled beneficiaries across districts did not wish the research team to contact them to invite them to the study. Once the implementers supplied the research team with the contact details of sampled beneficiaries who they had managed to contact, and who had agreed to be called to be invited to the study, almost every sampled beneficiary who was contacted agreed to participate. Of the 1260 AGYW beneficiaries, 515 were successfully contacted and consented to participate, resulting in a response rate of 23.8% varying by district from 16.1% to 35.0% (further details of the survey methodology are available elsewhere^2^).

In the AGYW survey, we investigated the effect of COVID-19 and the lockdown on AGYW mental health and household circumstances. Analysis of the survey data was conducted using StataMP 14.^4^ We described key variables with frequencies (n) and proportions (%), overall and stratified by age group. The total population frequency and proportions were calculated with sample weights that were representative of the district and age group to which the AGYW belonged. Variables assessed in the survey (presented in Table 2) included:

- During lockdown, me or my family experienced financial (money) problems
- During lockdown I worried that my food would run out because of a lack of money
- During lockdown did you go a day and night without eating because of lack of food?
- During lockdown, did your relationships with your family (those who you live with) get worse?
- During lockdown, did you become more distressed and anxious?
- During lockdown, was there more violence in your home?
- During lockdown, was it harder to get the emotional support you need from people who usually support you?
- During lockdown, did you feel more worried about being physically abused by people who are close to you?
- During lockdown, did you feel more worried about being emotionally abused by people close to you?
- During lockdown, did you feel more worried about being sexually abused?

### Qualitative component

In the qualitative study component we conducted telephonic in-depth interviews (IDIs) with fifty [50] AGYW between the ages of 15 and 24 years (not the same AGYW who had participated in the survey), at which point it was determined that a sufficient level of data saturation had been reached. Semi-structured interview guides framed discussions, outlining key topics for discussion. Audio recordings were directly translated from their original language into English and reviewed by interviewer/s for accuracy. Qualitative data were coded by a team of three analysts, assisted by NVivo 12 software, and following a cyclical process of iterative thematic analysis. A set of pre-determined deductive code types were reflexively refined to reflect emerging topics during preliminary analysis; analysis presented in this paper was based on the key pre-determined deductive codes “COVID-19”, and “Mental Health”. Through collaborative interpretation, analysts engaged in data immersion, re-examining data at different stages in the process, documenting reflective thoughts and sharing growing insights during regular discussions. The use of analytic memos created an important extra level of narrative, providing an interface between participants’ data, researchers’ interpretations and wider theory.

Surveys and interviews were conducted by a team of female researchers, representing fluency in the languages spoken in the sampled districts. Interviewers received training in human subject research ethics, adolescent-responsive phone interview skills, and on the study specific methods, protocols and tools. Research ethical approval to conduct this study was granted by the SAMRC Research Ethics Committee (EC036-9/2020). A study team member contacted each of the AGYW telephonically to invite her to participate in the study and administered the consent process. If the AGYW was under 18 years of age, parental/caregiver consent was obtained prior to conducting the assent process with the AGYW. We invited consenting AGYW to participate in the survey or an interview in their language of choice. Consenting information was presented to participants in a way that was easy to understand and appropriate to the participants’ education level. Each participant received ZAR 100.00 (US$ 7.00) reimbursement.

### Findings

A total of 515 AGYW took part in the survey, comprising 264 in the 15–19 years age group, and 251 in the 20–24 age group. The qualitative study sample consisted of fifty AGYW, twenty in the 15–19 years age group, and thirty in the 20–24 age group.

Findings from both the survey and qualitative study components reveal the multitude of effects that the COVID-19 pandemic and the lockdown restrictions had on AGYW’s mental health, exacerbating situations of poverty, unemployment, food insecurity and other related mental health stressors. Narratives shared by respondents in the qualitative interviews described the way in which household loss of income, economic and food insecurity, fear of infection, and death of family members resulted in feelings of frustration, anxiety and depression.

**Table 2:** The impact of COVID-19 and the lockdown on the lives and mental health of AGYW in 6 South African districts, 2020–2021 Survey (n = 515)

	Never		Sometimes		Often	
	(Freq)	%	(Freq)	%	(Freq)	%
During lockdown, AGYW or her family experienced financial problems						
Total (N = 503)	142.3	28.3	235.6	46.9	125.1	24.9
15–19 (n = 255)	86.1	33.8	112.9	44.3	56.0	22.0
20–24 (n = 248)	48.4	19.5	132.6	53.5	67.0	27.0
During lockdown, AGYW worried that her food would run out because of a lack of money						
Total (N = 503)	123.8	24.6	246.3	49.0	132.9	26.4
15–19 (n = 255)	72.9	28.6	128.9	50.6	53.2	20.9
20–24 (n = 248)	49.2	19.8	120.0	48.4	78.9	31.8
During lockdown, AGYW went a day and a night without eating because of a lack of food						
Total (N = 503)	378.9	75.3	104.6	20.8	19.4	3.9
15–19 (n = 255)	193.4	75.8	48.3	18.9	13.3	5.2
20–24 (n = 248)	187.8	75.7	57.0	23.0	3.2	1.3
During lockdown, AGYW's relationship with her family (those with whom she lives) became worse						
Total (N = 502)	360.9	71.9	114.3	22.8	26.9	5.4
15–19 (n = 254)	184.4	72.6	54.4	21.4	15.2	6.0
20–24 (n = 248)	174.9	70.5	61.2	24.7	12.0	4.8
During lockdown, AGYW became more distressed and anxious						
Total (N = 503)	156.3	31.1	240.8	47.9	105.9	21.1
15–19 (n = 255)	87.0	34.1	123.8	48.6	44.2	17.3
20–24 (n = 248)	60.5	24.4	118.2	47.7	69.4	28.0
During lockdown, AGYW reported that there was more violence in her home						
Total (N = 504)	430.9	85.5	63.8	12.7	9.3	1.9
15–19 (n = 256)	220.4	86.1	31.7	12.4	3.9	1.5
20–24 (n = 248)	209.8	84.6	33.5	13.5	4.7	1.9
During lockdown, AGYW reported that it was harder to get the emotional support that she needed						
Total (N = 503)	246.8	49.1	190.5	37.9	65.7	13.1
15–19 (n = 256)	131.7	51.5	91.4	35.7	32.9	12.8
20–24 (n = 247)	114.4	46.3	103.0	41.7	29.6	12.0
During lockdown, AGYW felt more worried about being physically abused by people who are close to her						
Total (N = 503)	438.7	87.2	48.0	9.6	16.2	3.2
15–19 (n = 255)	216.6	84.9	32.3	12.7	6.1	2.4
20–24 (n = 248)	223.1	90.0	15.8	6.4	9.1	3.7
During lockdown, AGYW felt more worried about being emotionally abused by people who are close to her						
Total (N = 504)	389.8	77.3	89.8	17.8	24.4	4.9
15–19 (n = 256)	204.0	79.7	40.1	15.7	11.9	4.6
20–24 (n = 248)	176.8	71.3	58.7	23.7	12.5	5.0
During lockdown, AGYW felt more worried about being sexually abused by people who are close to her						
Total (N = 504)	470.0	93.3	28.5	5.7	5.5	1.1
15–19 (n = 256)	240.4	93.9	11.1	4.3	4.5	1.8
20–24 (n = 248)	228.9	92.3	17.3	7.0	1.8	0.7

### Socio-economic mental health stressors

Many AGYW participants (69%) reported they had become more distressed and anxious (sometimes or often) during COVID-19 and the lockdown; with more AGYW in the 20–24 year group reporting distress and anxiety (75.7%) than AGYW in the 15–19 year group (65.9%) (Table 2). In the survey, 71.8% of all participants reported that she or a family member experienced financial problems (sometimes or often) during COVID-19 and the lockdowns; reporting considerably higher amongst young women aged 20–24 (80.5%) than among adolescent girls aged 15–19 (66.3%). Narratives of increased stress, anxiety, financial and food insecurity also emerged in the qualitative data. A common experience during lockdowns related to household loss of income, and employment, with many households relying on social grants to survive.

*COVID affected us a lot because my father lost his job and we were struggling… sometimes we would sleep on an empty stomach. Even the money for the two children that we get (child grant) was not helping us because we had to use it for everything. The worse part was that we had to buy (infant feeding) formula and diapers with it. We were greatly affected. (AGYW 20-24 years)*

Most AGYW (75.4%) reported concerns about food running out (sometimes or often) due to lack of money during COVID-19 and the lockdown, with reporting higher in the older age group (80.2%) than the lower age group (71.5%). A quarter (24.7%) of all AGYW aged 15–24 reported they had gone a day and night without food sometimes or often due to lack of money during COVID-19 and the lockdown (Table 2).

During interviews, respondents spoke of the ways in which loss of income at the household level brought about situations of hunger and feelings of stress and desperation.

*People in our community are poor… they are starving, you see children who are hungry… children stealing at the shops… when you ask the child what is wrong she will say “my mother is not working, my father is not working, I haven’t eaten since last night”… there is financial strain. (AGYW 20-24 years)*

Those AGYW who had lost parents had to deal with grief, as well as anxiety about survival and meeting basic needs.

*I get stressed… how will I cope without parents (who have died), where will I get food, how am I going to pay rent? (AGYW 15–19 years)*

Desperation was enhanced for AGYW who experience unintended pregnancies, HIV infection and those who face challenges feeding their babies or children, dependent on the child support grant.

*The issues we face here are issues with the environment we live in… We are dying… We are dying of HIV, poverty, depression and some young people are taking their lives… you can’t sleep on an empty stomach… rather a person would just cut themselves with a razor blade and die… Out of my friends, many of them have died of suicide because they were depressed and not knowing how to survive… A girl gets unplanned pregnancy by mistake… then she is expected to maintain the child with the R400 (US$ 28.00) which comes once a month, and the question is, can you feed a child with one formula for the whole month?... there is a lot of kids who are dying because of depression… We are dying, and the world will end up without the youth, because of the things we go through. (AGYW 20-24 years)*

### Family relationships and domestic violence

Regarding reporting on the impact of COVID-19 and lockdown on family relationships, 28.2% of all AGYW reported that relationships with family members had (sometimes/often) worsened (27.4% of 15–19 year olds, and 29.5% of 20–24 year olds) (Table 2). Some AGYW survey participants reported that since the pandemic and the lockdown, there was (sometimes/often) more violence in their home (14.6%), and that they were (sometimes/often) more worried about being physically abused (12.8%), emotionally abused (22.7%), or sexually abused (6.8%). In the qualitative study AGYW described situations in which some families, being confined together in small homes, combined with hunger, unemployment, fostered feelings of frustration and financial insecurity, and created tensions, exacerbating existing issues of gender-based violence, which also added to levels of fear, stress, anxiety, and other negative mental health outcomes. Loss of income and feeling of confinement also translated to feelings of anger, powerless and frustration, which at times manifested in physical abuse.

*With these restrictions and rules, there was a rise in gender based violence… we had to be at home most of the time… people were taking out their frustrations on their partners. (AGYW 20-24 years)*

For those AGYW living in homes where violence was an issue, lacking the ability to escape during lockdowns fostered fear of having to face abuse at home. For some school-going AGYW who come from abusive or unhappy families, school usually provides a respite from abusive parents and difficult situations at home, however with schools being closed, they were ‘stuck’ at home. Despite families being forced to spend more time together at home, respondents described emotional distancing between family members confined together, resulting in further loss of social connection and emotional support.

*In my family we were being distanced from each other at home… we weren’t like we usually were, each and every person would be in their room. (AGYW 20-24 years)*

More than half (51%) of AGYW reported they had found it (sometimes/often) harder to get the emotional support they needed during COVID-19 and the lockdown (53.7% in the 20–24 year old group and 48.5% in the 15–19 year old group). Qualitative study respondents articulated the view that the lockdowns had served to exacerbate existing social divisions, resulting in a lack of social support in the community setting.

*It kind of distances people… as a community we are not close, but it made it worse… people have a reason now to say that we can’t come together as a community because of COVID. It is has given them a reason to justify that. (AGYW 20-24 years)*

### Depression, frustration and loneliness

In the qualitative study, AGYW shared their sense of frustration and depression during the lockdown period, when movement was restricted and people had to stay at home.

*Covid-19 lockdown affected me a lot… I was not at peace… to wake up and do nothing is so frustrating, it affects the mind… you are always thinking. (AGYW 20-24 years)*

AGYW respondents shared narratives of the onset of depression under lockdown, describing feelings of being bored, frustrated, isolated, hopeless, and sad.

*Due to COVID everything stopped and everyone had to be indoors, locked up… it affected me in a very big negative way. In the beginning of course, when it first started it was all well and good but then it became really bad, because then I got into depression… I was always indoors with nothing to do, my brain was going crazy… I just kind of shut it down. In a negative way it affected me… just everything, the economy going down, people being retrenched at their workplaces… Even though I try and put a happy face… I am still sad. (AGYW 20-24 years)*

The sense of being trapped or stuck indoors with family, without anything to do, fostered boredom and household tension. This was coupled with the fear of leaving the house.

*The lockdown impacted me in a big way because we could not do anything and that put a lot of strain on us… we were scared to go out, or to do anything. (AGYW 15-19 years)*

Feelings of loneliness and isolation emerged as salient themes in the narratives of respondents, combined with a sense of disruption to normal social interactions and connections.

*It becomes lonely in the house… we cannot associate with other people, it’s hard, we can’t go to see people. We cannot do what we are used to doing… Before me and my friends were able to sit together and support each other… We had days that we usually spent together and have fun. We cannot do those things now because of COVID… we cannot meet and spend time together like before. (AGYW 15-19 years)*

### Fear

Fear was another salient theme in the qualitative data. AGYW described being scared of leaving home due to fear of both COVID-19 infection, and of police. But at the same time they know that staying at home means that there are no opportunities for making any money.

*People are afraid to walk on the streets because of COVID, and on the other side police are patrolling… people are complaining, saying “maybe let’s see if there’s a job opening somewhere”… you don’t have money… people are staying at home… but if you are staying at home there’s no money. (AGYW 20-24 years)*

Respondents shared narratives in which fear and anxiety were prominent emotions. One reason for anxiety cited by respondents related to worry about family members dying of COVID-19.

*You are always worried that maybe the people who are taking care of you will have get affected by COVID and die. (AGYW 20–24 years)*

For those AGYW whose parents were still working, there was fear that they would get infected, and bring the virus into the family and household.

*At the start of lockdown… I was stressed because we got information that it (COVID) is here and it kills people… my life started to change… when you walk around the street… if you’re not wearing a mask you get arrested… things were complicated… It was this corona virus every time on the news… the people who are positive have increased, others have passed away… and on the other hand my mother is going to work, so that was scaring me to think it is not safe… she will bring it (virus) here at home and… we all become positive (infected) at home. (AGYW 15-19 years)*

The fast-changing policies and evolving restrictions, including those related to school closures, meant that there were high levels of uncertainty and anxiety.

*COVID was affecting us… I was disturbed. Schools were opening and closing… that also disturbs your mind. Because you don’t know what to expect, we were always just waiting to see what was going to happen, so it disturbed the mind a lot. You can’t even have plans. (AGYW 15-19 years)*

## Hopelessness

Due to the multitude of challenges, exacerbated by the lockdown, some AGYW experienced a sense of hopelessness.

*Life is difficult… you see yourself losing hope. (AGYW 20–24 years)*

At times the hopelessness became overwhelming, and AGYW described reaching a point where they gave up hope.

*At the moment, to be honest… I have just given up hope… At this point… I don’t really know how I feel, there is so much going on, so many emotions that I am emotionally numb in a way. (AGYW 20-24 years)*

Fear and worry about the future was a prominent theme in AGYW narratives. Respondents described feeling that the dreams and aspirations they had prior to COVID-19 had been shattered.

*If Corona was not here, I can imagine how far I would have been now. I would not be in the place I am now, because of COVID, and that makes my heart sore. (AGYW 20-24 years)*

Feelings that the pandemic had changed the world, and concern that things would never be the same again, fuelled anxiety amongst AGYW.

*I am a bit anxious about my dreams if are they going to come true, looking at the rapidly high rate of deaths, and my question is, will I ever achieve all the things I want in life if people are dying left and right? (AGYW 20-24 years)*

Respondents felt that life had been disrupted, time wasted, and future plans had to be abandoned, which gave AGYW a feeling of being stuck.

*COVID affected me a lot because I had dreams, I wanted to fulfil, they are now stuck because I can’t carry on with my life as per norm… a lot of time has been wasted. (AGYW 15-19 years)*

### Resilience

Despite the multitude of hardships and mental health stressors, some of the AGYW respondents articulated emotional resilience, describing their coping mechanisms.

*It has shown me that, no matter what happens you have to push hard when you want to achieve something. Even though things are stuck at the moment, you have to tell yourself that you will do things bit by bit… I have that mind-set of doing something despite the challenges. (AGYW 15-19 years)*

Maintaining hope despite challenges and uncertainty was a key form of emotional resilience described by respondents.

*At the moment… I am scared but at the same time I have hope that the plans I have for this year, maybe can still be fulfilled… currently, I am nervous because I don’t know what to expect. (AGYW 15-19 years)*

*It’s my future that makes me worried… because it’s not clear and known as to what is going to happen… it’s only by having hope that everything will work out nicely… because it’s uncertain about what is going to happen in future. (AGYW 15-19 years)*

Resilience also manifested in feelings of acceptance and faith that everything will be ok, and that circumstances will improve.

*During times like this, it requires one to accept the situation because something that has a majority of people has no control… The best you can do is to follow what is being ordered until it gets better… to do what we are told to do. (AGYW 15-19 years)*

Strategies of resilience described by respondents were not just at the individual level; some AGYW spoke of their supportive social circumstances and sources of psychosocial support such as parents, grandparents, and faith groups. Mothers were commonly cited as support providers, helping AGYW cope.

*My mother she supports me with everything… whenever she sees that I’m not okay you will see by when she becomes sad she will ask me: “you’re not okay, talk to me, what’s happening?” (AGYW 20-24 years)*

*My mum is always there for me… Whenever I have a problem… I can to talk to her about issues that affect me. She will then come with a solution to help me out. Like in issues I had at school, she was there as my shoulder (support). (AGYW 15-19 years)*

## Discussion

In this study we examined the ways in which COVID-19 impacted mental health amongst AGYW in South Africa. Our study focused specifically on AGYW who lived in districts in South Africa that had been identified as high priority for health interventions, characterised by high HIV prevalence, high rates of teenage pregnancy, and disproportionately affected by socio-structural drivers of these. Prior to the pandemic, AGYW in these communities already faced a range of socio-economic, structural and environmental mental health stressors, which were further exacerbated by COVID-19. Findings showed how COVID-19 increased household financial strain and food insecurity and how these in turn resulted in increased stress, anxiety and feelings of desperation. AGYW reported worsening family relationships, and increased fear of domestic violence relating. COVID-19 control measures including lockdown and social gathering restrictions led to feelings of boredom and frustration, a sense of being trapped, isolated and lonely, combined with an underpinning of fear. Respondents described an overwhelming sense of hopelessness about their current situation, and the future. However, on a positive note, despite the multitude of hardships and mental health stressors experienced, some AGYW respondents articulated an emotional resilience, describing the ways in which they managed to cope in healthy ways, and retain hope.

There were noteworthy differences between age groups in terms of proportions of AGYW reporting experiencing financial problems, and worry about food running out due to lack of money, with those in the older age group more likely to experience these stressors, compared with the younger group. One reason for this could be that older AGYW take more responsibility for securing livelihoods in the home compared with the younger AGYW. When it came to being more worried about abuse, AGYW in the younger group were more worried about being physically abused by people close to them, compared with those in the older group, perhaps because they are more likely to be dependent on others in the home and therefore less likely to be able to protect themselves from physical abuse. In contrast with this, AGYW in the older group were more likely to report being worried about emotional abuse by people close to them, compared with the younger age group.

### Socio-economic mental health stressors

Our findings provide important evidence showing that among the multitude of environmental influences on mental health, the inability to fulfil one’s basic needs, having sufficient food in particular, is significantly associated with poor mental health. This aligns with pre-epidemic data on the association between hunger and food insecurity in adolescents and internalising behaviour problems such as depression and anxiety [15, 30]. Our findings are also consistent with the literature, showing that COVID-19 restrictions, whilst nationally applicable, have impacted South Africans in differing ways, disproportionately affecting the poorest, most marginalised and vulnerable in society due to socioeconomic realities such as inequality, poverty, violence, and rising unemployment [10, 31]. Evidence suggests that globally, the COVID-19 pandemic is affecting vulnerable groups, such as AGYW, disproportionally, further exacerbating pre-existing inequalities [13, 27]. High levels of inequality and poverty exist in South Africa, and the majority of South Africans live below the poverty line. Poorer communities have borne the brunt of COVID-19 lockdowns, with restrictions disproportionately affecting those families and individuals already living in poverty, increasing unemployment and food insecurity [11, 32, 33].

### Family relationships and domestic violence

Our findings add to emerging evidence that lockdown and economic strain have put families into an immediate state of distress, impacting negatively on relationships, and resulting in increased physical, sexual, emotional violence experienced by AGYW [18, 34–37]. As described by respondents in our study, family confinement during lockdown, in some cases led to a deterioration in family relationships, acting as a trigger for intrafamilial violence [18, 34]. It is possible that alongside increased gender-based violence, gendered power inequities have been enhanced, therefore exacerbating AGYW risks and vulnerabilities [38].

Some AGYW articulated the sense that instead of close proximity and spending more time together strengthening bonds between family members, the forced proximity led to family members emotionally isolating themselves, diminishing the availability of emotional support in the home environment. Narratives of tension at home, and fraught family relationships due to lockdown and family members feeling stressed, frustrated and confined in close quarters have also emerged in other South African studies [28, 37]. A lack of social connections and interaction fostered feelings of loneliness and isolation amongst AGYW in our study, with respondents reporting they had found it harder to get the emotional support they needed during the lockdowns. A recent study amongst workers in South Africa also found that social distancing had led to loneliness amongst adults [39]. Additionally, evidence suggests a link between experienced loneliness, “containment-related stress” related to stay-at-home lockdown restrictions, and mental health issues such as anxiety, depression and suicide ideation [2, 40]. Death of parents or caregivers also adds additional stressors to adolescents’ mental health, and can lead to increased risk behaviours [41]. Social isolation exacerbates the vulnerabilities of AGYW, and those living with abusive household members may have been at increased risk of interpersonal violence victimisation, including sexual violence and intimate partner violence [42]. With one of the world’s strictest lockdowns, South Africa’s physical, social, and economic restrictions, South Africans with and without existing mental health issues faced heightened levels of loneliness, depression, harmful substance use, and suicidal behaviour [10].

### Depression, frustration and loneliness

Our findings corroborate evidence emerging from South Africa, and globally, suggesting that adolescents and young people are experiencing increased levels of insecurity and anxiety [13]. Fear, worry and distress are normative responses to unprecedented uncertainties and difficulties [11, 20]. Our findings build upon recent research that demonstrates an association between higher reporting of depressive symptoms with factors including being female and residing in urban informal areas [13]. Prior to the COVID-19 epidemic, AGYW in South Africa, particularly those from low-resource communities, already faced substantial social adversities and related mental health challenges and stressors [1]. COVID-19 restrictions have disrupted normal day to day living, and have affected various aspects of young people’s physical, mental and social health, further compounding existing mental health stressors, adding feelings of social isolation, loneliness, uncertainty, anxiety and depression [9]. Evidence from just prior to the period in which we collected data for this study, demonstrates links between the economic shock that occurred in between February and April 2020 and a significant increase in depressive symptoms in South Africa between May and November 2020 [43, 44].

### Fear

As illustrated by our findings, there are a number of ways through which the COVID-19 and the lockdown has affected the mental health of AGYW in South Africa. Fear was a predominant emotion in the narratives of respondents in our qualitative study: fear of infection, fear for the health and safety of family, fear related to economic insecurity and future prospects, and fear of leaving home during lockdowns due to concerns about police brutality (during the hard lockdown, the South African police force and the military had been deployed to ensure compliance) [40]. Mental health and wellbeing are influenced by a multitude of factors, many of these are circumstantial, environmental, social, and interpersonal in nature [14]. As seen in our findings, and other recent research, fear, emotional distress, anxiety and uncertainty, and depression provoked by the COVID-19 pandemic are not only due to fear of infection and death, but also due to environmental and socio-economic stressors, food and income insecurity [27, 28, 43–45]. Even without the added stressors introduced by COVID-19 and lockdown, young people in South Africa face substantial social adversities and related mental health challenges due to a range of SRH, social, economic, environmental, physiological and interpersonal factors, however this burden has clearly increased [1, 13].

### Hopelessness

Respondents in our study described their emotions of hopelessness, with a sense of defeat about their futures. AGYW in our study articulated feelings of being stuck and frustrated, having their dreams for the future shattered, and losing hope. Other emerging evidence suggests that the feeling of being stuck, and having your life put on hold is a common experience amongst adolescents and young people in the context of COVID. Two recent studies amongst young South Africans have also revealed experiences of depression and distress, alongside feelings of helplessness, being ‘stuck’ or ‘trapped’, being overwhelmed, and a sense of lacking control over their own lives [28, 46]. Similarly to AGYW in our study, anxiety and uncertainty about the future, leading to a sense of hopelessness and despondence was also found amongst South African university students [46]. Feelings of defeat and hopelessness about educational and career prospects, accompanied by negative perceptions of self-potential, apathy and avoidance of opportunities, are not only distressing due to economic anxiety, but could link to psychological distress related to loss of purpose and agency, and are likely to make it more difficult for young people to escape circumstances of poverty and adversity [2, 4]. To mitigate these impacts, efforts need to be made to create an enabling environment to foster hope amongst adolescents and young people in South Africa [30].

### Resilience

Despite the various hardships and challenges described by AGYW in our study, some respondents also demonstrated emotional resilience and described ways in which they managed to cope with the challenges they faced. Psychological resilience amongst adolescents and young people, their ability to manage feelings of stress, address stressors they encounter, or gain a feeling of coping with their circumstances, is critical to protecting and buffering the impact on mental health [1]. Not all adolescents are successful in coping with the stress they face, however, as illustrated by the AGYW in our study, some do demonstrate an ability to cope. Individual, relational and contextual factors, such as poverty, geopolitics, age, gender, mental health and parenthood, shape an individual’s ability to buffer against material and psychosocial shocks, and constrain their ability to cope [28]. Experiences of an overwhelming feeling of hopelessness, and the strain that the COVID-19 pandemic and lockdown has placed on people’s ability to maintain a hopeful outlook are common themes emerging in current research [27]. It is critical to respond to these feelings of hopelessness, to mitigate their negative effects on mental health, and to tap into adolescents’ and young people’s emotional resilience capabilities to maintain hope in the face of challenges.

In our study, AGYW who demonstrated resilience described their own ways of coping including taking things one step at a time, maintaining hope against the odds, and simply accepting their situation and being patient that things will improve. Respondents also described sources of psycho-social support which enabled them to cope, such as parents, grandparents, and community/faith based groups. Evidence from previous disaster situations shows that some adolescents have efficient and effective coping strategies, and have capacities for resilience; these existing coping mechanisms should be bolstered and strengthened [18]. While recognising the creativity, resilience and agency of South African adolescents and their communities, the contextual factors that limit their ability to exercise agency, build resilience and cope in the time of COVID-19 should be considered [28].

As a theoretical lens through which to examine our findings, we drew on the syndemic theory, referring to the co-occurrence and interaction of social conditions and inequities, combined with the notion of intersectionality. Intersectional vulnerabilities, risks, and disadvantages based on characteristics such as gender, age and SES status – in the case of AGYW in our study – are produced and continually reproduced in a dynamic relationship, with a cumulative effect that exceeds the sum of the detrimental effects derived from each single characteristic [47]. Applying an intersectional lens to the concept of syndemics, which often focuses on individual vulnerabilities, adds a consideration the intersections at which an individual exists – gender, age, SES status – helping to account for a diversity of experiences within a population, explaining the resilience demonstrated by some AGYW in our sample [48]. In this way, intersectionality and the syndemic theory can help to explain the ways in which intersectional vulnerabilities and risks combine to influence the ways different AGYW experience vulnerability and resilience [47, 49]. Our data and that of other studies, suggest that SES status was an important determinant for how COVID-19 lockdowns were experienced, with poverty as a vulnerability factor that exacerbated pre-existing inequalities, negatively impacting on the wellbeing of poorer households. SES inequalities are associated with inequities in SRH, education, and mental health among adolescents; as shown in our findings and corroborated in the literature, there are distinct and particular vulnerabilities that AGYW in South Africa experience. In this sense, the impacts of COVID-19 restrictions and lockdowns added additional burdens that interact with pre-existing and co-occurring vulnerabilities faced by AGYW, to create yet another dimension of vulnerability and risk [47, 49].

Several strengths and limitations of our study should be noted. A limitation in the design of the survey was reflected in the final survey sample response rate, which was lower than expected. The sampling strategy depended on participants being contactable via cell-phone, meaning that those AGYW who were not contactable are likely to be different to, and possibly more vulnerable than those who had access to working cell-phones, and this may have introduced a bias in the study findings. Regarding the methodology of conducting the survey and interviews remotely, there are both strengths and limitations. Remotely conducted interviews, while limiting the opportunity for interviewers to build rapport with participants, can allow for increased disclosure of sensitive behaviours and reduced social desirability bias [50]. An additional limitation relates to the fact that data was collected during a specific time period, reflecting AGYW experiences and emotions during the five months of data collection, and thus may not reflect the changes during subsequent COVID-19 waves and restrictions. A strength of our study, enabled by the remote data collection methodology, was that the sample was drawn from a large geographic area spanning six provinces, and therefore offered a level of generalisability of AGYW from diverse communities across South Africa.

## Conclusions and recommendations

The focus of our research was on AGYW from communities in South Africa identified as being priority areas for health interventions. Various psycho-social risk factors already disproportionally affect the mental health of AGYW in these communities; the COVID-19 pandemic intersects and overlaps with these pre-existing social and environmental factors. COVID-19 and the lockdown exacerbated pre-existing mental health stressors, and led to increased levels of anxiety, emotional distress, and other negative emotions amongst AGYW in these communities. It is critical to consider the mental health of AGYW in these high-priority communities, even more so given the intersections between mental health, and sexual and reproductive health amongst AGYW, and the links between psychological distress and increased risk behaviours. Interventions are needed to support the psychological and emotional wellbeing of AGYW, and to bolster their coping mechanisms; these interventions need to be responsive to the pandemic environment.

Our findings add to emergent evidence revealing increased rates of stress, panic disorder, anxiety, depression and feelings of loneliness, resulting from a multitude of COVID-19 related mental health stressors including fear of infection, concerns about family health, income and job losses, experiences of illness, concern about lack of access to basic needs, food insecurity, death of loved ones, restriction of movement, social isolation through loss of physical and social interactions, and increases in domestic violence [9, 12, 13, 25, 30, 41, 51–53].

Despite the high burden of mental health challenges, policies and services specifically targeted at adolescents and young people are woefully inadequate in South Africa [54, 55]. This was the case even prior to the COVID-19 pandemic, but this lack has been accentuated by the recent crisis. Additionally, the distribution of mental health resources across the country is markedly inequitable, particularly in the public sector health system [55]. In order to mitigate the negative impacts and buffer against additional COVID-19-specific stressors, the South African government urgently needs to recognise child and adolescent mental health services as a health priority, and develop appropriate, innovative, cost-effective, scalable, evidence-based systemic or multi-level interventions, to promote the mental health of adolescents and young people [9, 48, 54]. The South African government’s efforts to provide financial relief thus far may not be sufficient; for example, other research indicates that implementation of social protection measures including the COVID-19 grant were not reaching those in need equally, particularly for women, and people living in rural areas. Additional tailored outreach to ensure uptake may be needed to fully address the differential impact of hunger, unemployment, poverty, depression, and gender-based violence on certain groups that are risk factors for poor mental health [26].

Critically, given the immense and unprecedented scale of social disruption caused by the pandemic, mental health interventions need to be responsive to the specific social determinants in each context, especially those exacerbated by COVID-19, in order to achieve their intended impacts [20]. The COVID-19 restrictions in South Africa, while aiming to protect the health of the population, have exacerbated social inequalities, underlying socio-structural vulnerabilities and life challenges for the most vulnerable groups in South Africa [27], such as the AGYW in the communities in which this study was conducted. Mental health is not possible without having basic needs met. Therefore, strengthened social protection responses and social safety nets and the provision of relief packages to meet basic needs and alleviate acute distress and respond to food insecurity and hunger during COVID-19 lockdowns, combined with loss of income for families are critical to mitigate the effects of the pandemic [14, 28]. In light of findings suggesting that government relief did not always reach those in need, showing that socio-economic hardship adversely affects AGYW mental health adds weight to current debates around the ways in which COVID-19 has strengthened calls for a basic income grant in South Africa, with unconditional and universal coverage.

Key to mitigating mental health stressors is addressing structural drivers such as unemployment and food insecurity [15, 30]. Economic empowerment programmes for AGYW may help to reduce mental health stressors; combined with efforts to build resilience to maintain young people’s confidence and motivation to persevere with education and employment opportunities [4, 9]. Interventions to promote resilience can be effective, and therefore opportunities to promote resilience should be harnessed [56]. Resilience can be fostered through multiple pathways; social capital, psychosocial support and self-help interventions and “strength-based prevention programmes”, may also help to enhance AGYW’s resilience and coping skills [14, 24]. Important considerations for the integration of mental health into social protection interventions include targeting the most vulnerable AGYW, and the provision of soft skills modules and mental health interventions that could help to boost resilience [2].

Community-based mental health care could be harnessed by increasing investment in, and support for community-based civil society organisations who understand and can respond to the needs of the communities in which they operate and the impact of the pandemic therein [55]. Using platforms such as schools and community-based services in the provision of basic mental health support programmes could help to increase access to mental health care, and in turn mainstream and promote mental health during periods of heightened adversity, buffering against pandemic related stressors, and bolstering cohesion and resilience at the community level, even in the absence of physical proximity [14, 27, 30, 39, 55]. Integrating mental health care support into school-based services may be a cost-effective opportunity for promoting mental wellbeing and resilience amongst adolescents and young people [57]. Addressing the mental health treatment and care gap in South Africa, particularly in rural and/or resource restricted communities, through building skills and capacity of lay mental health providers, and frontline workers including community health workers and teachers, and improving access to mental health literacy resources could help to promote resilience and increase access to necessary care and support [9, 30, 57].

Other recommendations include the provision of accessible and data free Mobile health applications, digital technologies, virtual support solutions and online platforms to deliver brief evidence-based psychological interventions for young people with symptoms of depression and anxiety, and provide psycho-social support through online/digital and telephonic counselling services, and through community safe spaces to reduce isolation and address gaps in psycho-social support [2, 39]. Whilst digitally based interventions can be effective and support improved coverage of mental health support and care, potential harms of digital technology for adolescents and young people need to be considered, and efforts need to be made to ensure equity in access [57].

There should be increased investment and research into innovative ways to ensure AGYW access to health, social protection and educational interventions during situations like the pandemic. In addition, interventions to provide parenting support and investments in family strengthening could fill additional gaps in mental health support for young people, especially in resource restricted settings [41]. Schools and educational institutions can also play a critical role in identifying and assessing learners in need of support and care, making referrals to community-based workers and the Department of Social Development [30]. In summary, a multi-sectoral and coordinated response is needed to address unemployment and food insecurity, and reduce key drivers of poor mental health [30].

## Data Availability

The datasets used and/or analysed during the current study are available from the corresponding author on reasonable request.

## Footnotes

1 When excess deaths are factored in, it has been calculated that nearly 300,000 people have died from COVID-19 in South Africa, thrice the official figure of 97,000, according to reporting by the SAMRC (https://www.samrc.ac.za/reports/report-weekly-deaths-south-africa).

2 https://doi.org/10.1016/S0140-6736(21)02,143-7.

3 https://www.samrc.ac.za/intramural-research-units/healthsystems-herstory.

4 StataMP 14, StataCorp, Texas, USA.
